# Cortisol-Induced Chromatin Remodeling and Gene Expression in Skeletal Muscle of Rainbow Trout: Integrative ATAC-Seq and RNA-Seq Analysis

**DOI:** 10.3390/ijms26136079

**Published:** 2025-06-25

**Authors:** Rodrigo Zuloaga, Camila Garrido, Luciano Ahumada-Langer, José Luis Galaz, Giorgia Daniela Ugarte, Alfredo Molina, Juan Antonio Valdés

**Affiliations:** 1 Programa de Doctorado en Biotecnología, Facultad de Ciencias de la Vida, Universidad Andres Bello, Santiago 8370186, Chile; rodrigo.zuloaga.r@gmail.com; 2 Laboratorio de Biotecnología Molecular, Departamento de Ciencias Biológicas, Facultad de Ciencias de la Vida, Universidad Andres Bello, Santiago 8370146, Chile; c.garridopino@uandresbello.edu (C.G.); lucianofranco.a@gmail.com (L.A.-L.); jlgalaz@uchile.cl (J.L.G.); giorgia.ugarte@unab.cl (G.D.U.); amolina@unab.cl (A.M.); 3 Interdisciplinary Center for Aquaculture Research (INCAR), Concepción 4030000, Chile; 4 Programa de Fisiología y Biofísica, Facultad de Medicina, Instituto de Ciencias Biomédicas (ICBM), Universidad de Chile, Santiago 8380453, Chile; 5 Instituto de Ciencias Biomédicas, Facultad de Ciencias de la Salud, Universidad Autónoma de Chile, Santiago 8320000, Chile; 6 Centro de Investigación Marina Quintay (CIMARQ), Universidad Andrés Bello, Quintay 2340000, Chile

**Keywords:** cortisol, stress response, rainbow trout, ATAC-seq, RNA-seq

## Abstract

Cortisol, the main glucocorticoid in teleost, plays a central role in mediating the physiological response to stress by regulating metabolism, immune function, and growth. While its transcriptional effects are well known, its role in modulating chromatin accessibility in fish skeletal muscle remains poorly understood. In this study, we investigated the epigenomic and transcriptomic changes induced by cortisol in a juvenile rainbow trout’s (*Oncorhynchus mykiss*) skeletal muscle using ATAC-seq and RNA-seq. Fish were treated with a single intraperitoneal dose of cortisol (10 mg/kg) or vehicle, and muscle samples were collected 3 h post-treatment. ATAC-seq analysis revealed a total of 163,802 differentially accessible regions (DARs), with an important enrichment of open regions near transcription start sites and promoters. A total of 1612 and 1746 differentially accessible genes (DAGs) were identified in the cortisol and control groups, respectively. Motif enrichment analysis identified 89 transcription factors to be significantly enriched, among which key stress-responsive regulators such as Fos, AP-1, FoxO1/3, Mef2a/b/c, Klf5/10, and ATF4 were prominently represented. RNA-seq analysis identified 4050 differentially expressed genes (DEGs), with 2204 upregulated genes involved in autophagy, mitophagy, and FoxO signaling, while 1864 downregulated genes were enriched in spliceosome and chromatin remodeling pathways. Integrative analysis revealed 174 overlapping genes between ATAC-seq and RNA-seq datasets, highlighting pathways linked to autophagy and ATP-dependent chromatin remodeling. Four selected DEGs (*sesn1*, *sesn2*, *cullin3*, *samtor*) were validated by qPCR, showing high concordance with transcriptomic data. These findings provide new insights into cortisol-mediated regulation of chromatin dynamics and gene expression in teleost skeletal muscle and underscore the importance of epigenetic mechanisms in fish stress responses.

## 1. Introduction

Aquaculture is crucial for meeting the rising global need for seafood, contributing significantly to food security, economic development, and sustainable environmental practices [1]. Among aquaculture sectors, salmonid farming stands out as one of the most significant contributors to global aquatic food production [2]. The farming of rainbow trout (*Oncorhynchus mykiss*), known for its adaptability, rapid growth, and high nutritional value, represents a crucial segment of salmonid aquaculture, particularly within freshwater systems and across a wide range of production scales [3,4]. In 2022, rainbow trout aquaculture achieved a total production of 959,600 tons, with an estimated market value of USD 3.2 billion [5]. The remarkable production levels in aquaculture have been made possible through the adoption of intensive farming practices [6,7]. However, such conditions characterized by high stocking densities, frequent handling, temperature variability, and fluctuating water quality expose fish to stress, which can impair health, weaken immune function, and reduce growth performance, ultimately challenging the long-term sustainability of aquaculture operations [8,9].

Fish, like other vertebrates, possess highly conserved physiological mechanisms to cope with environmental and husbandry-related stressors [10]. The primary stress response is mediated by the activation of the hypothalamus–pituitary–interrenal (HPI) axis, leading to the release of cortisol, the main glucocorticoid in teleost [11]. Cortisol plays a central role in restoring homeostasis by modulating metabolism, osmoregulation, immune function, and energy allocation [12]. Cortisol binds to intracellular glucocorticoid receptors (GRs), modulating gene expression by interacting with glucocorticoid response elements (GREs) in target gene promoters [13]. Chronic elevation of cortisol due to prolonged stress has been shown to inhibit growth by redirecting energy toward stress adaptation mechanisms, impairing muscle protein synthesis and increasing catabolic activity [14]. Skeletal muscle, which accounts for approximately 60% of a fish’s body mass, is a key determinant of growth and metabolic efficiency [15]. Unlike mammals, teleost fish exhibit indeterminate muscle growth throughout their lifespan, making muscle development a critical factor for aquaculture productivity [16]. Cortisol influences the balance between protein synthesis and degradation, acting as a key regulator of anabolic pathways such as the growth hormone/insulin-like growth factor-1 (GH/IGF-1) axis, and promoting catabolic processes through activation of the ubiquitin–proteasome and autophagy–lysosome systems [17,18].

Beyond its immediate physiological effects, cortisol is also implicated in epigenetic regulation, influencing gene expression through mechanisms such as DNA methylation and histone modifications, which can have long-lasting impacts on gene expression and physiological adaptation to stress [19,20,21]. Evidence from Atlantic salmon (*Salmo salar*) has shown that early-life stressors, such as cold shock and air exposure, can alter DNA methylation and gene expression patterns, highlighting the role of environmental conditions in shaping the epigenome [22]. Whole-genome bisulfite sequencing (WGBS) studies have further demonstrated stressor-specific methylation changes associated with immune function and transgenerational plasticity [23,24]. Another important epigenetic mechanism, widely studied in plant and mammals, is the remodeling of chromatin accessibility [25]. However, the pathways through which cortisol modulates chromatin accessibility in the skeletal muscle remain largely unexplored in teleost. Advances in next-generation sequencing (NGS) technologies, such as Assay for Transposase-Accessible Chromatin with high-throughput sequencing (ATAC-seq), provide a powerful approach to mapping genome-wide chromatin accessibility changes [26]. This technique enables the identification of transcription factor binding sites and regulatory elements associated with stress responses, offering new insights into the epigenetic regulation of muscle growth under aquaculture-relevant stress conditions [27]. While ATAC-seq has been applied to study chromatin accessibility in various biological contexts, including fish gill tissue and metamorphosis in flatfish [28,29], its application in stress-induced tissues remodeling remains scarce [30]. Despite the growing evidence of cortisol’s epigenetic effects in vertebrates, the dynamic regulation of chromatin accessibility in fish skeletal muscle remains largely uncharacterized [31]. Understanding these epigenetic mechanisms could lead to the identification of biomarkers for stress resilience, facilitating selective breeding strategies for improved aquaculture productivity [32]. Additionally, characterizing the chromatin landscape in response to cortisol-mediated stress may provide novel insights into how stress impacts muscle growth at the molecular level, ultimately contributing to the development of more sustainable fish farming practices. This study aims to characterize the effects of chronic stress and cortisol on chromatin accessibility in rainbow trout skeletal muscle using ATAC-seq. By integrating transcriptomic and epigenomic analyses, we seek to unravel the regulatory networks underlying stress-induced muscle remodeling, providing essential information for enhancing aquaculture efficiency and fish welfare.

## 2. Results

### 2.1. Analysis of Chromatin Accessibility Induced by Cortisol in Rainbow Trout Skeletal Muscle

Juvenile rainbow trout were stimulated with a single dose of cortisol at physiological concentrations (10 mg/kg). To prevent an increase in cortisol production due to handling stress, individuals were pre-treated with metyrapone (1 mg/kg). Three hours after treatment, plasma cortisol (230.5 ± 34 ng/mL) and glucose (53.3 ± 9 mg/dL) levels significantly increased in the cortisol-treated groups compared to the control (Table S1). Treatment with metyrapone did not modify baseline cortisol and plasma glucose (Table S1). ATAC-seq analyses for control and cortisol were performed with three biological replicates at each condition. A total of 551,091,086 raw reads were obtained with an average length of 151 bp corresponding to 6 cDNA libraries. After filtering out low-quality reads and adaptor sequences, 550,474,588 clean reads were acquired with an average length of 100 bp. Remarkably, 94% of reads were mapped to the rainbow trout reference genome (Table 1).

**Figure 1:**
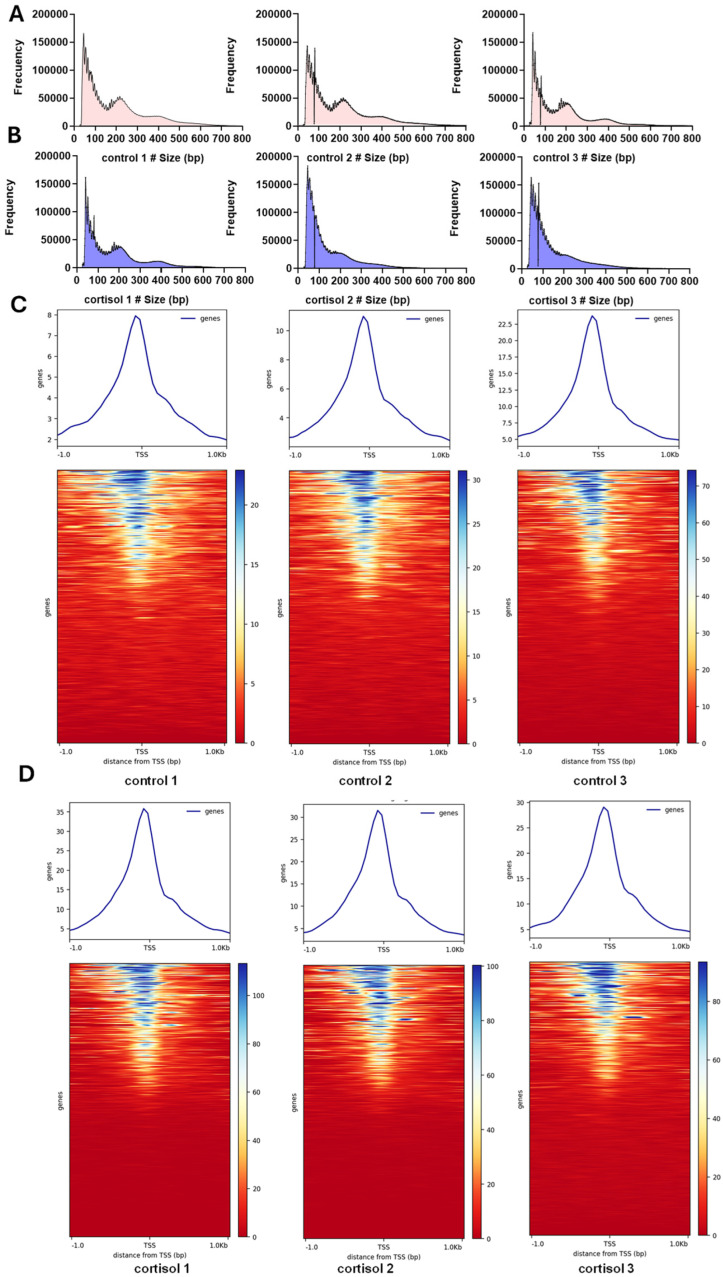
ATAC-seq data. (**A**) Fragment length distribution in control groups. (**B**) Fragment length distribution in cortisol groups. (**C**) Heatmap of read distribution across gene bodies and peaks in control groups. (**D**) Heatmap of read distribution across gene bodies and peaks in cortisol groups.

All libraries exhibited a multimodal distribution of fragment lengths. Specifically, the first peak, observed at around 50 base pairs (bp), corresponded to Tn5 transposase insertions within nucleosome-free regions. The second peak, slightly below 200 bp, reflected insertions around a single nucleosome. A third peak, approximately 400 bp in size, indicated Tn5 insertions spanning two adjacent nucleosomes, while a fourth peak, around 600 bp, corresponded to insertions at around three nucleosomes (Figure 1A,B). This fragment length pattern is characteristic of the periodic organization of nucleosomes within chromatin and validates the quality of the ATAC-seq libraries generated. Most of the ATAC-seq peaks were enriched 1 kb upstream and downstream of the transcription initiation site, which indicated that open regions of chromatin were involved in transcriptional regulation. The heatmap showed that ATAC-seq accessibility for cortisol groups was slightly higher than for control groups (Figure 1C,D), suggesting a dynamic regulation of chromatin accessibility induced by cortisol.

**Figure 2:**
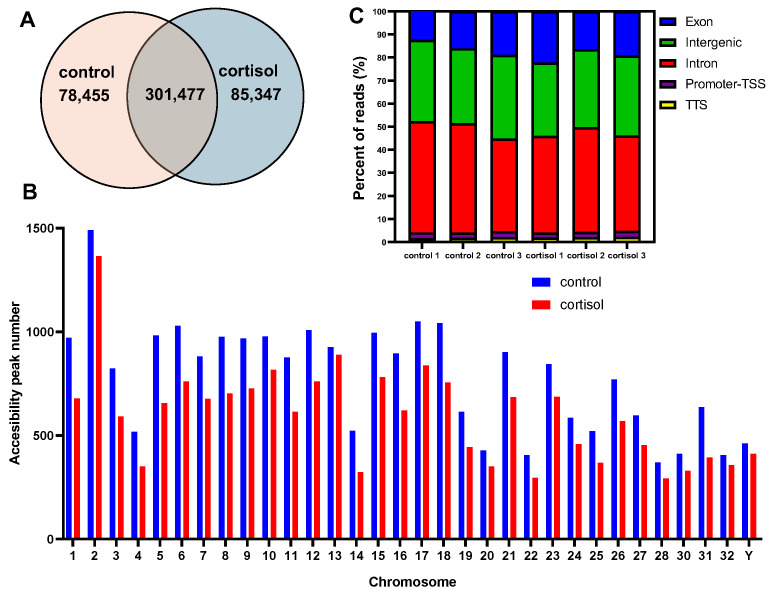
Analysis of chromatin accessibility. (**A**) Venn diagram showing the peak overlap between control and cortisol groups. (**B**) Chromosomal distribution of accessibility peaks number. (**C**) Genomic distribution of accessibility peaks in functional regions which include promoter–TSS, intergenic, exon, intron, and TTS.

We identified 78,455 and 85,347 specific accessible chromatin peaks or DARs (Differentially accessed regions), and 301,477 common peaks in control and cortisol groups, respectively (Figure 2A). The distribution of chromosomal peaks in the rainbow trout genome is illustrated in Figure 2B. The open chromatin region maps at the chromosome level in the whole genome showed most of the regions on each chromosome. To annotate the genomic distribution of open chromatin peaks, they were assigned to genome-wide functional regions, including Transcription Termination Sites (TTS), intergenic, promoter–transcription start site (TSS), intron, and exon (Figure 2C).

**Figure 3:**
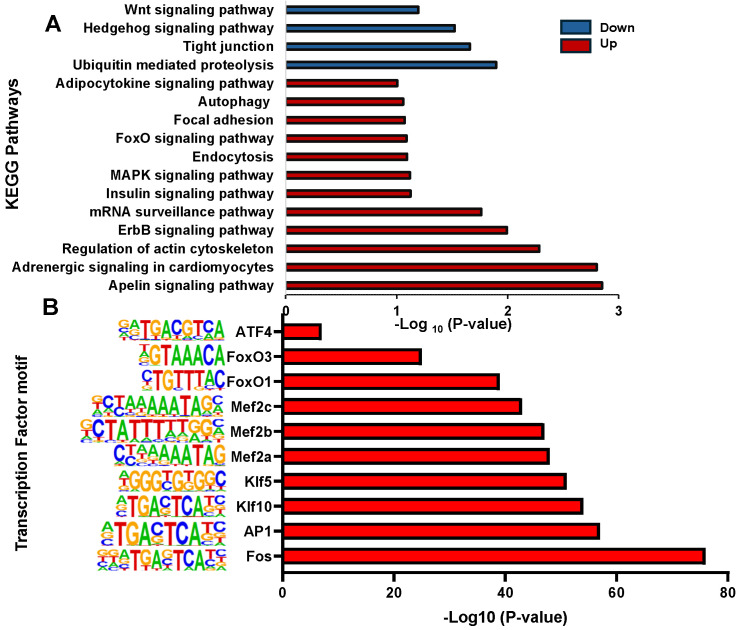
KEGG enrichment analysis and motifs analysis of genes associated with differential chromatin accessibility. (**A**) KEGG pathway enrichment analysis of DAGs (**B**) Enriched transcription factor binding motifs in DAGs (*p* < 0.01).

Most peaks were mapped at intergenic, exon, and intron regions. Annotating each peak revealed that the different peaks in the cortisol group correspond to 1612 DAGs and in the control group correspond to 1746 DAGs. To explore the potential functions of these genes, we conducted GO and KEGG enrichment analysis. We found that the biological processes in GO analysis were mainly related to protein phosphorylation, regulation of transcription by RNA polymerase II, and calcium ion transmembrane transport, among others (Table S2). Molecular functions were mainly enriched in protein binding, metal ion binding, and transcription factor binding (Table S3). Cellular components were mainly enriched in the cytoplasm, nucleus, and mitochondrion (Table S4). In addition, the KEGG enrichment analysis revealed that upregulated DAGs were enriched in pathways associated with Apelin signaling pathways, Adrenergic signaling in cardiomyocytes, and the regulation of the actin cytoskeleton (Figure 3A). Downregulated DGAs were enriched in pathways associated with Ubiquitin-mediated proteolysis, Tight junction, and Hedgehog signaling pathways. Other interesting pathways enriched are the endocytosis, autophagy, and MAPK signaling pathway. HOMER software v5.1 was used to analyze transcription factor binding motifs at different peaks in rainbow trout and compare them with mammalian transcription factor databases. We identified 89 transcriptions factors significantly enriched (*p* < 0.01). Among those, the top ten transcription factors annotated were Fos, AP1, Klf10, Klf5, Mef2a, Mef2b, Mef2c, FoxO1, FoxO3, and ATF4 (Figure 3B).

### 2.2. Analysis of Gene Expression Induced by Cortisol in Rainbow Trout Skeletal Muscle

To determine the transcriptional response associated with cortisol administration, we sequenced the RNA from the skeletal muscle of rainbow trout with samples obtained three hours after treatment. A total of 335,693,902 reads were obtained corresponding to 6 cDNA libraries. After discarding adapters and low-quality reads, we obtained 335,623,996 clean reads for RNA-seq analysis. The average mapping rate was 87.9% in the rainbow trout genome (Table 2).

**Figure 4:**
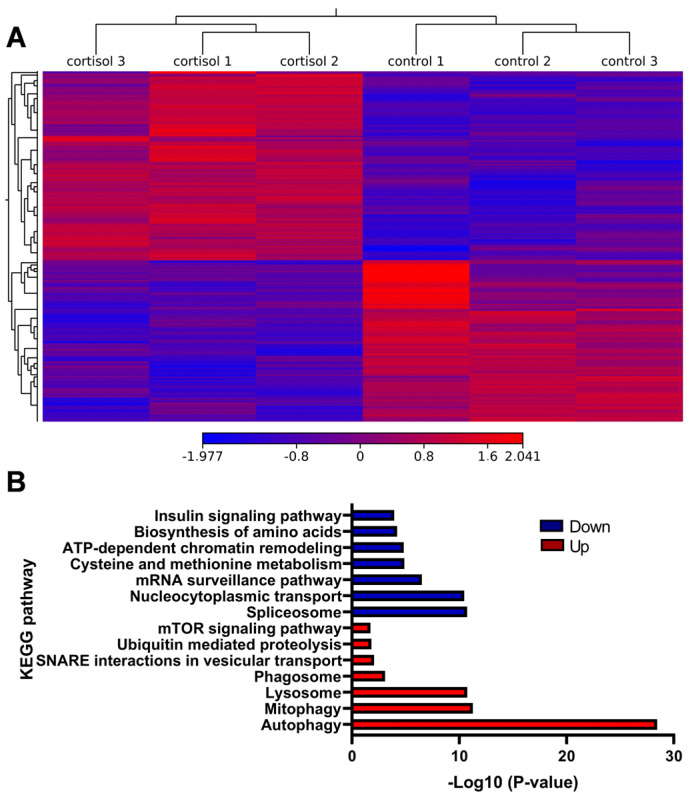
Analysis of RNA-seq. (**A**) Heatmap of the differentially expressed genes in the control and cortisol groups (**B**) KEGG enrichment analysis of DEGs.

We identified 4050 differentially expressed genes (DEGs) between control and cortisol groups, comprising 2204 upregulated and 1846 downregulated genes (Figure 4A). GO and KEGG enrichment analyses were performed. For the upregulated DEGs, the most highly enriched Gene Ontology (GO) terms in biological processes (BP) included autophagy of mitochondrion, autophagosome assembly, and piecemeal microautophagy of the nucleus (Table S5). In the molecular function (MF) and cellular component (CC) categories, the most enriched GO terms were protein binding (Table S6) and autophagosome (Table S7), respectively. KEGG pathway analysis revealed significant enrichment in autophagy, mitophagy, and lysosome pathways. Other interesting enriched KEGG pathways include Ubiquitin-mediated proteolysis, mTOR signaling pathway, and FoxO signaling pathway (Figure 4B). The downregulated DEGs were enriched in biological processes such as protein folding, protein phosphorylation, and mRNA splicing via the spliceosome (Table S8). GO terms for downregulated genes were associated with RNA binding (MF) (Table S9) and the nucleolus (CC) (Table S10). The KEGG pathway indicated significant enrichment in the spliceosome, nucleocytoplasmic transport, and mRNA surveillance pathways. Other interesting enriched KEGG pathways include ATP-dependent chromatin remodeling, cysteine and methionine metabolism, and biosynthesis of amino acids (Figure 4B).

### 2.3. Integrative Analysis of ATAC-Seq/RNA-Seq and Validation

**Figure 5:**
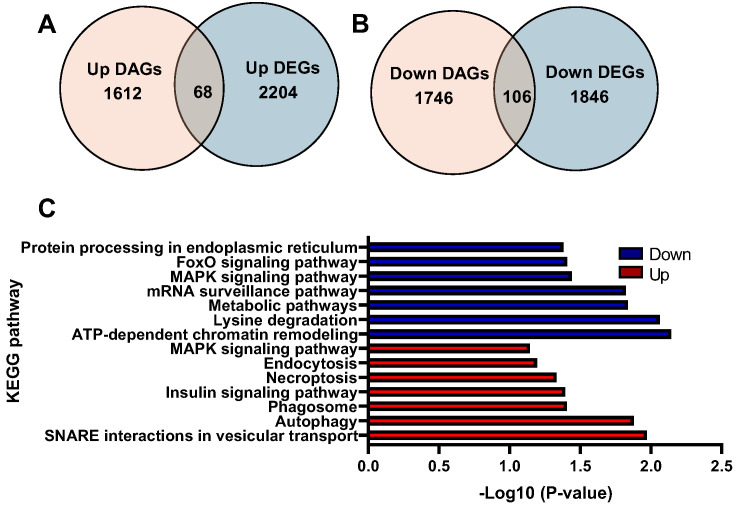
Integrative analysis of ATAC-seq and RNA-seq: (**A**) Venn diagram of upregulated DAGs and upregulated DEGs. (**B**) Venn diagram of downregulated DAGs and downregulated DEGs. (**C**) KEGG enrichment analysis of common upregulated and downregulated DAGs and DEGs.

Integrative findings of ATAC-seq and RNA-seq showed an overlap of 68 genes between 2204 upregulated DEGs and 1612 upregulated DARs (Figure 5A). GO enrichment analysis of these genes revealed that chromatin remodeling, vesicle docking, and vesicle fusion were overrepresented in the biological process (Supplementary Table S11). In cellular component and molecular function, histone acetyltransferase complex and histone H3K36 demethylase activity were significantly enriched, respectively (Supplementary Tables S12 and S13, respectively). The KEGG pathways analysis revealed that SNARE interactions in vesicular transport, autophagy, and phagosome were highly represented (Figure 5C). On the other hand, there was an overlap of 106 genes between 1846 downregulated DEGs and 1746 downregulated DARs (Figure 5B). GO enrichment analysis of these genes revealed that cellular response to hypoxia, anterograde axonal transport of mitochondrion, and regulation of DNA-templated transcription were highly represented (Supplementary Table S14). In cellular components and molecular functions, mitochondrion and ATP binding were significantly enriched, respectively (Supplementary Tables S15 and S16, respectively). The KEGG pathways analysis revealed that ATP-dependent chromatin remodeling, lysine degradation, and metabolic pathways were overrepresented (Figure 5C).

**Figure 6:**
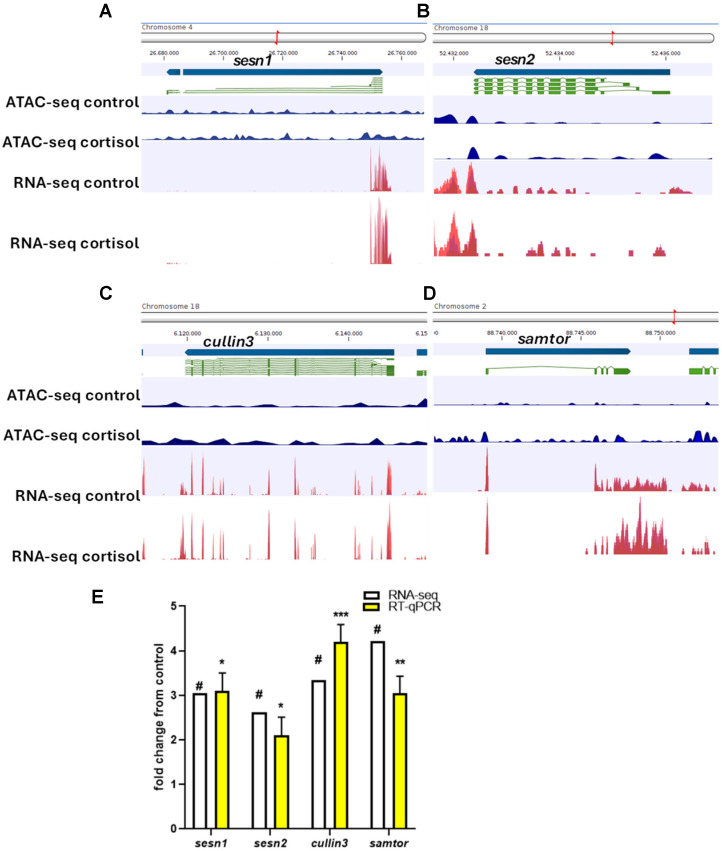
(**A**) Peak diagram for ATAC-seq and RNA-seq signal for *sesn1*. (**B**) Peak diagram for ATAC-seq and RNA-seq signal for *sens2*. (**C**) Peak diagram for ATAC-seq and RNA-seq signal for *cullin-3*. (**D**) Peak diagram for ATAC-seq and RNA-seq signal for *samtor*. (**E**) Genes selected for the RT-PCR validation were *sesn1*, *sens2*, *cullin-3*, and *samtor*. For RNA-seq, in white, “#” indicates a log2 fold-change ≥ 2.0 and FDR < 0.05. For RT-qPCR, in yellow, relative expression was normalized against *fau* and actβ. The results are expressed as means and + standard errors (*n* = 5 per treatment). Differences between control and cortisol groups are shown in * *p* < 0.05, ** *p* < 0.01 and *** *p* < 0.001.

To visualize the relationship between chromatin accessibility and gene expression, track list tools of CLC genomics workbench v23.0 was used to integrate the ATAC-seq and RNA-seq signaling of the genes related to autophagy and phagosome. *Sesn1* (Figure 6A), *sesn2* (Figure 6B), *cullin3* (Figure 6C), and *samtor* (Figure 6D) exhibit significant differential expression and differential chromatin openness in cortisol groups. For validation, we selected these genes for RT-qPCR analysis. Our findings demonstrate a strong correlation (r = 0.9246) between gene expression levels obtained through RNA-seq and qPCR. (Figure 6E).

## 3. Discussion

Understanding how cortisol modulates skeletal muscle physiology is essential to improving fish welfare and productivity in aquaculture [33,34]. While the transcriptional actions of cortisol via the glucocorticoid receptor (GR) have been extensively documented [35,36], its potential role in shaping the chromatin landscape in teleost skeletal muscle remains largely unexplored. This study provides the first genome-wide analysis of cortisol-induced changes in chromatin accessibility in rainbow trout (*O. mykiss*) skeletal muscle, integrating epigenomic (ATAC-seq) and transcriptomic (RNA-seq) approaches to unravel the molecular mechanisms underlying stress-induced muscle remodeling. This study complements previous research conducted by our group, which has described cortisol-mediated epigenomic effects mediated by DNA methylation in skeletal muscle at 7 days post-administration [19], as well as the chronic effects of cortisol under crowding stress conditions after 30 days [20]. In the present study, we focused on the early effects of cortisol on chromatin remodeling using an experimental approach previously described [37,38]. To achieve this, rainbow trout were pre-treated with metyrapone, an inhibitor of endogenous cortisol synthesis induced by handling stress, followed by administration of a single dose of 10 mg/kg cortisol. As expected, cortisol treatment significantly elevated circulating cortisol and glucose levels in physiological range, confirming effective stimulation of the stress axis.

ATAC-seq data revealed increased chromatin accessibility in the cortisol-treated group, particularly in promoter and transcription start site (TSS) regions, consistent with active transcriptional regulation. Similar observations have been reported in mammalian in vitro models. For instance, studies using the A549 cell line (human lung epithelial cells) demonstrated that treatment with glucocorticoids such as cortisol or dexamethasone induces early chromatin opening as early as 4 h post-treatment, as revealed by ATAC-seq [39]. Comparable results have also been described in other cell types, including macrophages [40] and murine mammary epithelial cell lines [41]. In fish, there are relatively few reports addressing the effects of cortisol or stress on chromatin remodeling using ATAC-seq. For example, a study on zebrafish (*Danio rerio*) demonstrated that chronic cortisol exposure during early development leads to neuroendocrine dysregulation in adulthood [31]. In another study conducted in channel catfish (*Ictalurus punctatus*), the effects of cortisol on hepatic response to *Aeromonas hydrophila* infection were examined using RNA-seq and ATAC-seq [30]. However, both studies focused on medium- to long-term responses, leaving open the question of how cortisol affects chromatin accessibility at early time points. Despite the central role of skeletal muscle in growth, metabolism, and stress adaptation in teleost, its chromatin dynamics remain poorly understood, with most available data limited to transcriptional outcomes. This underscores the necessity of using ATAC-seq to uncover the cis-regulatory architecture and early epigenomic changes driven by cortisol in muscle tissue, similar to others analysis in fish [42]. The presence of periodic nucleosome patterns and high-quality metrics, such as enrichment of open chromatin in regulatory regions, validated the reliability of the ATAC-seq libraries, and is similar to others studied in mammals’ skeletal muscle [43,44]. We found 85,347 and 78,455 differentially accessible regions (DARs) homogeneously distributed across all chromosomes, similar to other studies [45]. We also determined that differential accessions to chromatin were mainly distributed in intergenic regions, exons and introns, although a significant number were found in promoter regions close to the transcription start site [46]. Importantly, we identified more than 3300 DAGs in control and cortisol groups, suggesting that cortisol triggers widespread chromatin remodeling in skeletal muscle. Functional enrichment analysis of these DAGs revealed that pathways such as mRNA surveillance, actin cytoskeleton regulation, adrenergic signaling, endocytosis, autophagy, and MAPK signaling were overrepresented. Interestingly, similar patterns of chromatin accessibility associated were recently reported in goat bronchial epithelial cells infected with *Pasteurella multocida* [47]. Motif enrichment analysis revealed that stress-responsive transcription factors such as Fos, AP-1, Klf5/10, Foxo1/3, Mef2a/b/c, and ATF-4 are likely central regulators of the observed chromatin changes. Many of these transcription factors are known to participate in catabolic and oxidative stress pathways, and their enriched presence near accessible chromatin regions points to a cortisol-dependent activation of muscle remodeling programs [48,49]. Foxo1 and Foxo3 positively regulates starvation-induced muscle atrophy in fine flounder (*Paralichthys adspersus*) [50]. Foxo transcription factors promote muscle atrophy by activating genes involved in protein degradation, such as atrogin-1 and MuRF1 from the ubiquitin–proteasome system. Their activation is enhanced under stress or fasting in teleost, when IGF-1/Akt signaling is suppressed, allowing Foxo to enter the nucleus and induce catabolic gene expression [51]. Krüppel-like factor 10 (Klf10) has been described as regulators of muscle wasting in cancer (cachexia) [52], and Klf5 was upregulated in atrophying myotubes as an early response to dexamethasone or simulated microgravity in vitro [52]. Krüppel-like factor promotes proteolysis in fish skeletal muscle by upregulating components of the ubiquitin–proteasome and autophagy pathways, while suppressing anabolic signaling [53]. ATF-4 is a member of the basic leucine zipper (bZIP) transcription factor superfamily. Studies on mammals have shown that ATF4 can form heterodimers with CCAAT/enhancer-binding protein β (C/EBPβ), and this complex mediates muscle atrophy by inducing genes involved in protein degradation and oxidative stress response [48]. ATF-4 modules autophagy and endoplasmic reticulum stress during hepatic lipotoxicity in zebrafish (*D. rerio*) [54].

RNA-seq analysis supported these findings by identifying over 4000 DEGs in response to cortisol. Upregulated genes were predominantly enriched in autophagy, mitophagy, and lysosome pathways—hallmarks of catabolic responses in skeletal muscle. This is in agreement with previous studies reporting that prolonged cortisol exposure activates proteolytic systems such as the ubiquitin–proteasome and autophagy–lysosome pathways, resulting in muscle wasting [17,18]. Concurrently, downregulated genes were enriched in spliceosome function, chromatin remodeling, and amino acid biosynthesis, further supporting the suppression of anabolic and transcriptional machinery under stress. Regarding early changes in gene expression, the results obtained are fully consistent with the genomic effects of cortisol previously described for both glucocorticoid (GR) and mineralocorticoid (MR) receptors in rainbow trout skeletal muscle [37,38]. The integrative analysis between ATAC-seq and RNA-seq datasets identified hundreds of overlapping genes with coordinated changes in chromatin accessibility and transcript abundance. To validate our results, we selected 4 genes that showed a positive correlation in their chromatin accessibility and gene expression, and are also involved in the negative regulation of TORC1 signaling (*samtor*, *sesn1*, *sesn2*) and protein ubiquitination (*cullin-3*). TORC1 (Target of Rapamycin Complex 1) is a highly conserved signaling hub that regulates cell growth, metabolism, and protein synthesis in response to nutrients, energy status, and stress [55]. In skeletal muscle, TORC1 activity promotes anabolic processes such as protein translation and inhibits autophagy. Its dysregulation under stress conditions, including glucocorticoid exposure, can lead to impaired muscle growth and metabolic imbalance [56]. The *samtor* gene encodes a key sensor of intracellular S-adenosylmethionine (SAM) levels and functions as a negative regulator of TORC1 signaling [57]. SAMTOR binds to the GATOR1 complex in a SAM-dependent manner, thereby modulating mTORC1 activity in response to cellular methylation status. Through this mechanism, *samtor* links nutrient sensing to the regulation of autophagy, growth, and metabolism, particularly under stress or nutrient-deprived conditions [58]. To date, there are no reports on the regulation of *samtor* expression in fish mediated by cortisol or stress. The *sesn1* and *sesn2* genes encode members of the Sestrin family of stress-inducible proteins that play crucial roles in cellular homeostasis [59]. Both genes are involved in the negative regulation of the mTORC1 signaling pathway and are key mediators of autophagy activation in skeletal muscle under stress conditions such as oxidative damage, stress, and nutrient deprivation [60]. The overexpression of *sesn1* has been reported in tambaqui (*Colossoma macropomum*) exposed to the insecticide malathion [61]. On the other hand, no reports are currently available regarding *sesn2* expression in teleost, although its crucial role in dexamethasone-induced muscle atrophy has been demonstrated in mice [62]. The *cullin-3* gene encodes a core component of the Cullin-RING E3 ubiquitin ligase complex, which plays a central role in targeting specific proteins for ubiquitination and subsequent proteasomal degradation [63]. In skeletal muscle, *cullin-3* is involved in regulating protein turnover, redox balance, and cellular responses to stress [64]. *Cullin-3* upregulation has been reported during acute cold stress in the skeletal muscle of zebrafish (*D. rerio*) [65]. Together, our findings demonstrate that cortisol not only regulates gene expression but also reprograms the chromatin architecture of skeletal muscle. The observed epigenetic modifications may contribute to long-lasting transcriptional memory of stress, a concept supported by recent evidence in mammals systems. The application of ATAC-seq in this context offers a powerful tool to identify regulatory elements and potential biomarkers of stress responsiveness, which could be leveraged in selective breeding programs to enhance fish robustness. Future studies should investigate the persistence and reversibility of these chromatin changes over time and explore their functional relevance under chronic stress or commercial aquaculture conditions. Moreover, integrating additional epigenetic layers such as histone modifications or DNA methylation would provide a more comprehensive understanding of stress-induced regulatory networks in fish muscle.

## 4. Materials and Methods

### 4.1. Experimental Protocol

The study was approved by the bioethics committee of the Andrés Bello University and the National Commission for Scientific and Technological Research of the Chilean government (protocol code 012/2023) and adhered to animal welfare procedures and the approved protocol. Juvenile rainbow trout (*Oncorhynchus mykiss*; 9.70 ± 2.6 g) were obtained from Pisciculture Río Blanco (V Region, Los Andes, Chile). Fish were maintained under natural environmental conditions, with a temperature of 14 ± 1 °C and a light:dark photoperiod of 12:12 h. They were fed daily with commercial pellets, with feeding suspended 24 h prior to the in vivo experiments. Experiments were carried out in 100 cm high tanks with a water turnover rate of 3.5 L/min, ensuring an oxygen concentration of 7 mg/L. Three tanks contained 5 individuals each, corresponding to non-manipulated group; three tanks contained 5 individuals each, corresponding to control group; and three tanks contained 5 individuals each, corresponding to cortisol group. For treatments, control group and cortisol group fishes were anesthetized using benzocaine (25 mg/L) and administered an intraperitoneal injection of metyrapone (1 mg/kg; Sigma-Aldrich, St. Louis, MO, USA). Following a 1 h pre-treatment, the control group received a vehicle solution (DMSO in PBS 1X), while the cortisol group received a single dose of cortisol (10 mg/kg; Sigma-Aldrich, St. Louis, MO, USA). After 3 h of incubation, all groups were euthanized with an overdose of benzocaine (300 mg/L). Blood samples were collected from the caudal vein using 1 mL syringes preloaded with heparin (10 mg/mL). Plasma was separated by centrifugation at 5000× *g* for 10 min at 4 °C, flash-frozen in liquid nitrogen, and stored at −80 °C for subsequent analysis. Samples of myotomal skeletal muscle were excised from the epaxial region, ground in liquid nitrogen, and subsequently stored at −80 °C. Plasma cortisol concentrations were determined using the Cayman cortisol assay kit (Cayman Chemical, Ann Arbor, MI, USA), and glucose levels were measured with a glucose assay kit (Abcam, Cambridge, UK).

### 4.2. ATAC-Seq Library Construction and Sequencing

Nuclei were isolated from skeletal muscle fibers following a standardized protocol. Briefly, tissues from three biological replicates were thawed on ice and dissected into fragments of approximately 2–3 mm. Mechanical dissociation was performed using scissors and a Dounce homogenizer. Tissue homogenates were washed with PBS 1x and treated with freshly prepared lysis buffer (10 mM Tris-HCl pH 7.4, 10 mM NaCl, 3 mM MgCl_2_, 0.1% NP40, protease inhibitor cocktail). Following the lysis, samples were centrifuged at 500× *g* for 5 min at 4 °C to pellet the nuclei. The supernatant was carefully removed, and the nuclei were washed once more with cold PBS 1X under the same centrifugation conditions. The nuclear suspension was then filtered through a 40 µm cell strainer to remove debris. After filtration, nuclei were stained with trypan blue, and 50,000 viable nuclei were counted for downstream applications. ATAC-seq libraries were prepared using the Zymo-Seq™ ATAC Library Kit (ZymoResearch, Orange, CA, USA) following the manufacturer’s instructions. Nucleolus were resuspended in 25 µL of ATAC-S Buffer, mixed with 25 µL of cold ATAC Lysis Buffer, and incubated on ice for 3 min. Nuclei were washed with 1 mL of cold ATAC Wash Buffer and pelleted by centrifugation at 1000× *g* for 9 min. Isolated nuclei were resuspended in a Tagmentation mix containing Pre-Tagmentation Buffer and Tn5 Enzyme and incubated at 37 °C for 30 min with shaking at 1000 rpm. Following Tagmentation, libraries were amplified by PCR using the ATAC Library PCR Mix and Unique Dual Index (UDI) primers provided in the kit. PCR cycling conditions were adjusted based on input amount and included an initial extension at 72 °C, followed by 8–10 amplification cycles. Amplified libraries were purified using DNA-Binding Buffer and DNA Wash Buffer, and DNA was eluted in 20 µL of Elution Buffer. Final libraries were quantified using Qubit dsDNA BR Assay (Invitrogen, Orange, CA, USA), assessed for fragment size distribution before sequencing using a capillary electrophoresis Fragment Analyzer Automated CE System (Advanced Analytical Technologies, Inc., Ames, IA, USA), and sequenced on Illumina NovaSeq6000 Illumina (150 bp PE) in Macrogen (Seoul, Republic of Korea).

### 4.3. ATAC-Seq Analysis

The initial processing of ATAC-seq data included quality assessment and adapter trimming of raw reads using TrimGalore with default parameters. Cleaned reads were subsequently aligned to the rainbow trout (*Oncorhynchus mykiss*) reference genome (OmykA_1.1, GCF_013265735.2), using Bowtie2 (v2.4.4) with default settings and very sensitive end-to-end parameters [66]. Bowtie2 alignment required the corresponding GTF annotation file to accurately map reads to genomic features. Following alignment, SAM files were converted to BAM format using SAMtools (v1.15.1) [67]. Low-quality and poorly aligned reads were filtered out, and BAM file indexing was performed to enable efficient data retrieval. Duplicate reads were identified and removed using Picard Tools (v2.27.4), based on mapping coordinates and read information, to ensure high-confidence downstream analyses. Peak calling to identify accessible chromatin regions was conducted using MACS2 (v2.2.7.1) with standard parameters. Peaks were called using a q-value threshold of <0.1 for weak signals and <0.05 for strong signals. Additional peak criteria included an enrichment fold-change > 1 and a normalized signal (pileup) >10 reads per million mapped reads. Genomic annotation of identified peaks was performed with HOMER (v4.11) [68]. Consensus peak calling across biological replicates was carried out using BEDTools (v2.30.0), highlighting peaks that overlapped between conditions, based on default overlap criteria. Aligned reads were then quantified against the consensus peak set using FeatureCounts (v2.0.1) [69]. For visualization, BAM files were converted into BigWig format using BedGraphToBigWig from the UCSC toolset [70]. Principal component analysis (PCA) and differential accessibility analysis were performed using DESeq2 (v1.38.0) to identify significantly accessible regions across experimental conditions [71]. Visualization and further inspection of aligned reads and accessible regions were conducted using the Integrative Genomics Viewer and CLC Genomics Workbench v23. The ID of differentially accessed genes were extracted and used as input to the DAVID GO and KEGG enrichment analysis [72]. To obtain the GO ID of each rainbow trout gene, we performed a BLASTx search against different fish databases, including Atlantic salmon (*S. salar*) and zebrafish (*D. rerio*), as previously described [37]. Standard settings for the DAVID analysis were as follows: gene count: 2 and ease: 0.1. The cut-off *p* value was 10^−2^. The KEGG pathways tool was used to identify enrichment pathways.

### 4.4. RNA-Seq Library Construction, Sequencing, and Analysis

RNA was extracted from the skeletal muscle of the control and cortisol groups using the RNeasy Mini Kit (Qiagen, Germantown, MD, USA) following the manufacturer’s recommendations. Total RNA integrity and quantity were assessed using the Agilent 2100 Bioanalyzer and Qubit RNA HS Assay Kit (Invitrogen, Carlsbad, CA, USA). Only RNA samples with RQN ≥ 8.5 were used in further analyses. RNA-seq libraries were prepared using the TruSeq RNA Sample Preparation Kit v2 (Illumina, San Diego, CA, USA) following the manufacturer’s protocol. Briefly, polyadenylated mRNA was isolated from 1 µg of total RNA using oligo(dT) magnetic beads. Purified mRNA was fragmented at 94 °C for 8 min and subsequently reverse-transcribed into first-strand cDNA using random hexamer primers and SuperScript II Reverse Transcriptase (Invitrogen, Carlsbad, CA, USA). Second-strand cDNA synthesis was performed to generate double-stranded cDNA. After purification, cDNA fragments were end-repaired, adenylated at the 3′ ends, and ligated to indexed sequencing adapters. Adapter-ligated fragments were enriched by PCR amplification (15 cycles) to complete library construction. Final libraries were quantified by qPCR and analyzed for size distribution using the Agilent Bioanalyzer (Agilent Technologies, Santa Clara, CA, USA) prior to sequencing. A total of 6 libraries were sequenced using a paired-end strategy (2 × 100 bp) with the NovaSeq6000 Illumina platform of Macrogen (Seul, Republic of Korea).

### 4.5. RNA-Seq Analysis

RNA-seq data were analyzed using CLC Genomics Workbench v23 (CLCQiagen, Germantown, MD, USA). Raw sequencing reads were first assessed for quality using the built-in quality control tools. Low-quality bases and adapter sequences were trimmed using the Trim Sequences tool with default settings: reads were trimmed based on a Phred quality score threshold of 20 and reads shorter than 50 nucleotides after trimming were discarded. Trimmed reads were mapped to the rainbow trout genome USDA_OmykA_1.1 (RefSeq GCF_013265735.2) using the RNA-Seq Analysis module which employs a median-of-ratios method to adjust for differences in library size and gene-specific biases. Mapping parameters were set as follows: mismatch cost = 2, insertion and deletion cost = 3, length fraction = 0.8, and similarity fraction = 0.8. Only uniquely mapped reads were retained for downstream analysis. Transcript quantification was performed using the “Expression Value Calculation” tool, generating values expressed as Transcripts Per Million (TPM) and Reads Per Kilobase of transcript per Million mapped reads (RPKM). Gene and transcript annotations were based on the corresponding GTF file associated with the reference genome. Differential gene expression analysis was conducted using the “Empirical Analysis of DGE” tool, which applies a negative binomial generalized linear model (GLM). We evaluated potential batch effects by performing principal component analysis (PCA) on our data. Genes with a false discovery rate (FDR) < 0.05 and an absolute fold-change ≥ 2 were considered significantly differentially expressed. The gene IDs of DEGs were extracted and used as input to the DAVID Gene Ontology (GO) enrichment analysis [70]. To obtain the GO ID of each rainbow trout gene, we performed a BLASTx search against different fish databases, including Atlantic salmon (*S. salar*) and zebrafish (*D. rerio*), as previously described [37]. Standard settings for the DAVID analysis were as follows: gene count: 2 and ease: 0.1. The cut-off *p* value was 10^−2^. The KEGG pathways tool was used to identify enrichment pathways.

### 4.6. Real-Time PCR Validation

Total RNA was extracted from skeletal muscle samples using the TRIzol reagent (Ambion, Carlsbad, CA, USA) following the manufacturer’s protocol. RNA concentration was determined using a NanoDrop spectrophotometer, and RNA integrity was assessed by electrophoresis on a 1.2% formaldehyde-agarose gel. For cDNA synthesis, 1 μg of total RNA was reverse-transcribed using the ImProm-II Reverse Transcription System (Promega, Madison, WI, USA) according to the supplier’s instructions. Primers for target gene amplification were designed using Primer3 software 4.1.0 accessed on 30 March 2024 (https://primer3.ut.ee/) and validated through NetPrimer 1.10 (http://www.premierbiosoft.com/netprimer/, accessed on 23 June 2025) and OligoAnalyzer 3.1 (https://www.idtdna.com/calc/analyzer, accessed on 23 June 2025). Quantitative PCR (qPCR) was performed on a Stratagene MX3000P system (Stratagene, La Jolla, CA, USA) using a reaction mix containing 7.5 μL of 2× Brilliant^®^ II SYBR^®^ Green Master Mix (Agilent Technologies, Santa Clara, CA, USA), 6 µL of 40-fold diluted cDNA, and 250 nM of each primer in a final volume of 20 μL. Negative controls included a no-template control (NTC) and a no-reverse transcriptase control (noRT) to check for contamination or genomic DNA amplification. The list of primer sequences used is provided in Supplementary Table S17. Amplification reactions were performed in triplicate under the following thermal cycling conditions: initial denaturation at 95 °C for 2 min, followed by 40 cycles of 30 s at 95 °C (denaturation), 30 s at 54–60 °C (annealing), and 30 s at 72 °C (extension). The specificity of amplification products was confirmed by a melting curve analysis at the end of each run. Relative gene expression analysis was performed using the 2^−ΔΔCT^ method [73], where expression levels were normalized to the vehicle-treated control group and expressed as fold-change. β-actin (*actβ*) and 40S ribosomal protein S30 (*fau*) were used as internal reference genes for normalization.

### 4.7. Statistical Analysis

All data were analyzed using one-way ANOVA and Tukey’s honestly significant difference (HSD) as a post hoc test, using the Graph Prism 8.0 software (GraphPad Software, Inc., San Diego, CA, USA). A probability level with a *p* value < 0.05 was used as the minimum to indicate the statistical significance.

## 5. Conclusions

This study provides the first integrative analysis of cortisol-induced chromatin remodeling and transcriptional reprogramming in the skeletal muscle of rainbow trout using ATAC-seq and RNA-seq. Our findings demonstrate that a single dose of cortisol triggers rapid and widespread changes in chromatin accessibility, particularly in promoter and regulatory regions, associated with the activation of key stress-related transcription factors such as Fos, AP-1, FoxO1/3, Mef2a/b/c, Klf5/10, and ATF4. These epigenomic changes were strongly correlated with transcriptional responses involving autophagy, mitophagy, ubiquitination, and suppression of anabolic and transcriptional pathways. Notably, the identification of stress-responsive genes such as *samtor*, *sesn1*, *sesn2*, and *cullin-3* reveals a mechanistic link between glucocorticoid signaling, TORC1 inhibition, and protein degradation pathways. This work advances our understanding of the molecular basis of cortisol action in teleost muscle and highlights the role of chromatin accessibility as a dynamic and early regulatory layer in the fish stress response. These insights have important implications for fish physiology and aquaculture, offering potential biomarkers and regulatory targets for improving stress resilience, growth performance, and animal welfare in farming systems.

## Figures and Tables

**Table 1:** Summary of the ATAC-seq data.

Name	Number of Reads	Avg. Length	Number of Reads After Trimming	Avg. Length After Trimming	Mapping Rate
Control 1	92,778,260	151	92,704,676	100	94.4
Control 2	91,565,508	151	91,452,112	100	93.2
Control 3	90,996,060	151	90,891,586	100	95.3
Cortisol 1	92,689,336	151	92,589,652	100	93.2
Cortisol 2	92,413,506	151	92,300,120	100	93.5
Cortisol 3	90,648,416	151	90,536,442	100	94.8
Average/total	551,091,086	151	550,474,588	100	94.1

**Table 2:** Summary of the RNA-seq data.

Name	Number of Reads	Avg. Length	Number of Reads After Trimming	Avg. Length After Trimming	Mapping Rate
Control 1	54,417,314	101	54,404,316	95.7	88.9
Control 2	61,509,806	101	61,498,920	95.6	87.3
Control 3	48,703,772	101	48,690,750	94.6	86.4
Cortisol 1	53,524,650	101	53,514,862	95.3	87.1
Cortisol 2	58,841,502	101	58,826,215	94.7	89.3
Cortisol 3	58,696,858	101	58,688,933	95.2	88.6
Total/average	335,693,902	101	335,623,996	95.5	87.9

## Data Availability

The raw read sequences acquired from sequencing were deposited in the Sequence Read Archive (SRA) under the following accession numbers: BioProject PRJNA1265628; BioSamples SAMN48602640, SAMN48602641, SAMN48602642, SAMN48602643, SAMN48602644, SAMN48602645. Accession SRX28866489, SRX28866490, SRX28866491, SRX28866492, SRX28866493, SRX28866494.

## Supplementary Materials

The following supporting information can be downloaded at: https://www.mdpi.com/article/10.3390/ijms26136079/s1.

## Abbreviations

The following abbreviations were used in this manuscript:

GR	Glucocorticoid receptor
MR	Mineralocorticoid receptor
DAGs	Differentially accessed genes
DARs	Differentially accessed regions
DEGs	Differentially expressed genes
qPCR	Real-time PCR
GO	Gene ontology
RQN	RNA quality number
